# Sentinel Listeriosis Surveillance in Selected Hospitals, China, 2013–2017

**DOI:** 10.3201/eid2512.180892

**Published:** 2019-12

**Authors:** Weiwei Li, Li Bai, Xiaochen Ma, Xiuli Zhang, Xinpeng Li, Xiaorong Yang, Jennifer Y. Huang, Séamus Fanning, Yunchang Guo

**Affiliations:** China National Center for Food Safety Risk Assessment, Beijing, China (W. Li, L. Bai, Y. Guo);; Beijing Center for Disease Control and Prevention, Beijing (X. Ma);; Henan Provincial Center for Disease Control and Prevention, Zhengzhou, China (X. Zhang);; Shandong Provincial Center for Disease Control and Prevention, Jinan, China (X. Li);; Sichuan Provincial Center for Disease Control and Prevention, Chengdu, China (X. Yang);; Centers for Disease Control and Prevention, Atlanta, Georgia, USA (J.Y. Huang);; University College Dublin, Dublin, Ireland (S. Fanning)

**Keywords:** listeriosis, surveillance, food safety, China, foodborne diseases, bacteria

## Abstract

During 2013–2017, a total of 211 cases of listeriosis were reported by 64 sentinel hospitals in China to a national foodborne disease surveillance network. The average case-fatality rate was 31.2% for perinatal cases and 16.4% for nonperinatal cases. Sequence types 87 and 8 were the most prevalent types.

Listeriosis is caused by the gram-positive bacterium *Listeria monocytogenes*, which is ubiquitous in the environment and a foodborne pathogen of importance to public health. Listeriosis occurs sporadically and mainly in high-risk groups, such as pregnant women, neonates, and immunocompromised and elderly persons (*1*). Although listeriosis occurs rarely in humans, it has a high case-fatality rate of 20%–50% (*2*). Nearly all reported listeriosis cases are transmitted to humans via food (*3*), and *L. monocytogenes* can grow at refrigeration temperatures, which makes it particularly challenging to control (*4*). 

In China, surveillance of *L. monocytogenes* in food products was launched in 2000 (*5*); however, as yet, listeriosis is not a notifiable disease in China. The National Foodborne Disease Surveillance Plan was implemented in 2011 (*6*,*7*). Human listeriosis surveillance was included as a special pilot project in 2013. We provide an overview of the listeriosis sentinel surveillance data for the period 2013–2017. We summarize the demographic and clinical characteristics of patients with listeriosis and analyze the prevalent sequence types (STs) of all identified isolates.

## The Study

In 2013, listeriosis surveillance started in 6 selected provinces in China. The target was to detect whether human listeriosis existed in China and to determine illness and death rates for listeriosis. In 2017, this pilot surveillance had expanded to 12 provinces with the additional objectives to investigate high-risk factors and detect potential outbreaks (Figure). A total of 78 sentinel hospitals were selected using convenient sampling: 40 general hospitals, 28 maternity hospitals, and 10 children’s hospitals.

**Figure:**
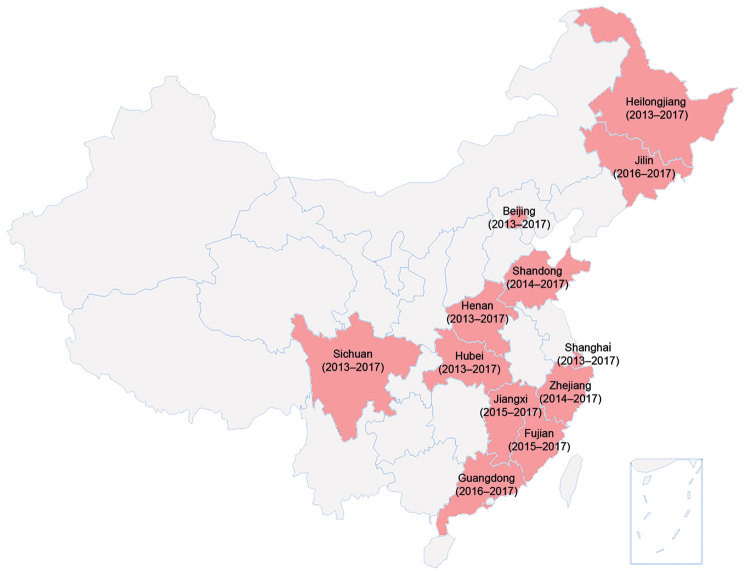
Geographic distribution of 12 selected provinces (red shading) included in human listeriosis surveillance, China, 2013–2017.

We defined invasive listeriosis as the isolation of *L. monocytogenes* from a normally sterile site (e.g., blood or cerebrospinal fluid) or products of conception (e.g., placental or fetal tissue) (*8*). Pregnancy-associated patients were considered perinatal case-patients, including pregnant women, fetuses, or infants <28 days of age; maternal–fetal infections were counted as a single case. We defined stillbirths and miscarriages as deaths, which were tallied in case-fatality rates. Pregnant women and neonates were the focused population groups, with immunocompromised and older adults also included. All demographic data, clinical manifestations, and laboratory tests were submitted to the China National Center for Food Safety Risk Assessment (CFSA) through the National Foodborne Disease Reporting System. All confirmed isolates were finally referred to CFSA for pulsed-field gel electrophoresis and whole-genome sequencing analysis through the National Molecular Tracing Network for Foodborne Disease Surveillance (TraNet).

During 2013–2017, a total of 211 listeriosis cases were diagnosed and reported by 64 sentinel hospitals, 138 (65.4%) perinatal cases and 73 (34.6%) nonperinatal cases. All case-patients were hospitalized; 55 deaths or fetal losses (case-fatality rate 26.1%) were reported, and 43 (78.2%) fatal cases occurred among fetuses and neonates. The average case-fatality rates were 31.2% (43/138) for perinatal and 16.4% (12/73) for nonperinatal cases. No maternal death was reported. Seventy-four (35.1%) case-patients acquired listeriosis in the summer (June–August).

Of the 138 perinatal infections, the median age of the mother was 29 years (range 20–41 years), and the median gestational age was 32 weeks (range 8–40 weeks). Preterm labor (<37 weeks gestational age) was reported in 63 (45.7%) pregnant women with listeriosis. Clinical signs in pregnant women included intrauterine infection, abortion, preterm labor, and influenza-like symptoms. Clinical manifestations and outcomes of infection in neonates included neonatal sepsis, asphyxia, pneumonia, meningitis, aspiration of amniotic fluid, meconium syndrome, and death.

Of the 73 nonperinatal infections, 45 (61.6%) cases were bloodstream infections such as septicemia and bacteremia, 20 (27.4%) were central nervous system infections, 6 (8.2%) were acute gastroenteritis, and 2 (2.7%) were focal infections. The median age of nonperinatal case-patients was 53 years (range 2 months–102 years); 22.9% were >65 years of age. The sex ratio was 1:1. Fifty-seven (78.1%) patients had positive blood samples, 11 (15.1%) had positive cerebrospinal fluid, and 15 (6.9%) were positive in other specimens, such as pleural effusion, cystic liquid, bone marrow, and feces (Table 1). The all-cause immunosuppression rate was 28.8% (21/73 cases). We detected the following underlying immunosuppression conditions: hematologic malignancy, systemic lupus erythematosus, chronic obstructive pulmonary disease, chronic kidney disease, liver disease, organ tumor, lung transplantation, and tuberculosis.

**Table 1:** Demographic data of 211 listeriosis case-patients reported by 64 sentinel hospitals, by risk group, China, 2013–2017*

Characteristic	Pregnancy-associated, no. (%)		Not pregnancy-associated, no. (%)		Total, no. (%)	
Total	138 (65.4)		73 (34.6)		211 (100.0)	
Sex						
F	138 (100.0)		36 (49.3)		174 (82.5)	
M	0		0		0	
Specimen source						
Blood	86 (62.3)		57 (78.1)		143 (67.8)	
CSF	8 (5.8)		11 (15.1)		19 (9.0)	
Other†	NA		5 (6.9)		5 (2.4)	
Product of conception	44 (31.9)		NA		44 (20.9)	
Death or fetal loss	43 (31.2)		12 (16.4)		55 (26.1)	

*CSF, cerebrospinal fluid; NA, nonapplicable. †Including pleural effusion, cystic liquid, bone marrow, and feces.

Of the reported listeriosis cases, 28.9% (61/211) were followed up with epidemiologic investigation, and 18.0% (11/61) yielded positive results for *L. monocytogenes* in suspicious food, chopping boards, refrigerator, or kitchen sinks. However, the pulsed-field gel electrophoresis patterns were not identical to those of clinical isolates, and >100 allele differences were found by using the core genome multilocus sequence typing (MLST) profile of 1,748 loci (*9*). These results showed no links between food, environmental, and clinical isolates.

A total of 116 isolates isolated during 2013–2016 were submitted to CFSA for whole-genome sequencing analysis: 108 from human listeriosis and 8 from the environment and suspicious food. The distribution of these 108 clinical *L. monocytogenes* clones was determined by MLST. A previous study reported that clonal complex (CC) 1, CC2, CC121, and CC155 were frequent clones in eastern Asia (*10*). We found that sequence type (ST) 87 (lineage I) and ST8 (lineage II) were the predominant STs; 15.7% of isolates were ST87 and 13.9% were ST8. The prevalences of ST87 in clinical isolates (*11*) and in domestic food products were also reported previously (*12*). ST87 was seldom linked to human listeriosis in other countries; only 2 outbreaks (both in Spain) were associated with ST87 strains (*13*). The most common PCR serogroups were IIb and IIa (Table 2). A total of 89 different core genome MLST types were identified as groups that differ by up to 7 allelic mismatches among the clinical isolates.

**Table 2:** Distribution of PCR serogroups and ST types identified among 108 isolates from listeriosis case-patients, by risk group, China, 2013–2017*

Characteristic	Pregnancy-associated, no. (%)		Not pregnancy-associated, no. (%)		Total, no. (%)	
Total	76 (70.4)		32 (29.6)		108 (100.0)	
PCR serogroups						
IIa	30 (39.5)		15 (46.9)		45 (41.7)	
IIb	32 (42.1)		13 (40.6)		45 (41.7)	
IVb	13 (17.1)		3 (9.4)		16 (14.8)	
IIc	1 (1.3)		1 (3.1)		2 (1.9)	
MLST types						
ST87	14 (18.4)		3 (9.4)		17 (15.7)	
ST8	11 (14.5)		4 (12.5)		15 (13.9)	
ST619	4 (5.3)		4 (12.5)		8 (7.4)	
ST155	2 (2.6)		4 (12.5)		6 (5.6)	
Other STs	45 (59.2)		17 (53.1)		62 (57.4)	

*MLST, multilocus sequencing typing; ST, sequence type.

## Conclusions

Our study describes epidemiologic characteristics of listeriosis from sentinel surveillance in China. An estimated 1,662 cases of listeriosis occur each year in the United States (*3*); a detailed analysis should be expedited in China to estimate incidence. The Universal Two-Child Policy was proposed and passed in 2015, which likely will increase the number of pregnancies and births in China and might therefore increase the incidence of listeriosis. 

This study has limitations (*1*). All cases came from sentinel hospitals but were not a complete picture of listeriosis occurrence because of the gradual increase of provinces included in surveillance (from 6 to 12 provinces), which meant the population served by selected hospitals could not be estimated accurately (*2*). All case-patients might be the most ill patients; cases might have been missed because those patients with milder illness might not go to the hospital and therefore will not be reflected in the data (*3*). The number of perinatal cases was nearly twice the number of nonperinatal cases, which cannot represent the actual illness and death rates because perinatal infection is given more attention in some sentinel hospitals (*4*). The case-fatality rates might be underestimated because all live-born infants, premature infants, and case-patients who did not complete follow-up surveillance were assumed to survive unless they were reported to have died.

In summary, health education and reasonable diet advice regarding listeriosis prevention should be provided to high-risk groups in China, and a focus on *L. monocytogenes* infection should be strengthened in hospitals. Moreover, *L. monocytogenes* is common in domestic food products in mainland China, especially in meat, poultry, seafood, and Chinese salad (*14*,*15*). An urgent need exists for improving surveillance of food and humans, exploring the mechanisms of pathogenesis, determining higher-risk foods, detecting potential outbreaks, and implementing control measures to protect vulnerable populations.
